# Majeed syndrome: description of a novel mutation and therapeutic response to bisphosphonates and IL-1 blockade with anakinra

**DOI:** 10.1093/rheumatology/kez317

**Published:** 2019-08-04

**Authors:** Noémi B A Roy, Ahmad I Zaal, Georgina Hall, Nick Wilkinson, Melanie Proven, Simon McGowan, Ria Hipkiss, Veronica Buckle, Akhila Kavirayani, Christian Babbs

**Affiliations:** 1 Department of Haematology, Oxford University Hospitals NHS Foundation Trust; 2 Oxford BRC Haematology Theme, University of Oxford; 3 Paediatric Rheumatology, Nuffield Orthopaedic Centre, Oxford University Hospitals NHS Foundation Trust, Oxford; 4 Children’s Hospital, Damascus University, Damascus, Syria; 5 Paediatric Haematology/Oncology Unit, Oxford University Hospitals, Oxford; 6 Paediatric Rheumatology, Evelina Children’s Hospital, Guy’s and St Thomas’ NHS Foundation Trust, London; 7 Molecular Haematology Laboratory, Oxford University Hospitals NHS Foundation Trust; 8 Computational Biology Research Group, MRC Weatherall Institute of Molecular Medicine, University of Oxford; 9 MRC Molecular Haematology Unit, MRC Weatherall Institute of Molecular Medicine, University of Oxford, Oxford, UK

**Rheumatology key message**

- Clinical variability in Majeed syndrome, favourable response to IL-1 blockade and recommendations for genetic screening.

Sir, Majeed syndrome, resulting from biallelic mutations in *LPIN2*, is a rare autosomal recessive autoinflammatory syndrome, originally described as a triad of chronic recurrent multifocal osteomyelitis (CRMO) or chronic non-bacterial osteomyelitis (CNO), congenital dyserythropoietic anaemia (CDA) and inflammatory neutrophilic dermatosis [1–3]. The CNO can affect various bones including the mandible, clavicle, spine and tibia [4]. Unlike sporadic CNO, which usually affects children between 4 and 15 years [4], CNO in Majeed syndrome is earlier in onset and is often refractory to conventionally prescribed NSAIDs and steroids [2]. The CDA reflects bone marrow ineffective erythropoiesis, with typical morphological abnormalities (e.g. binucleate erythroblasts, inter-nuclear bridging). The anaemia is microcytic and highly variable, ranging from sub-clinical to transfusion-dependent [2], and is distinct from anaemia of chronic disease. The inflammatory neutrophilic dermatosis or Sweet syndrome can present as pustulosis, plaques, nodules or ulceration [1–3] and has been recognized as being variably present in Majeed syndrome [2] while penetrance of the CNO and CDA has been described as complete.

**Fig. 1:**
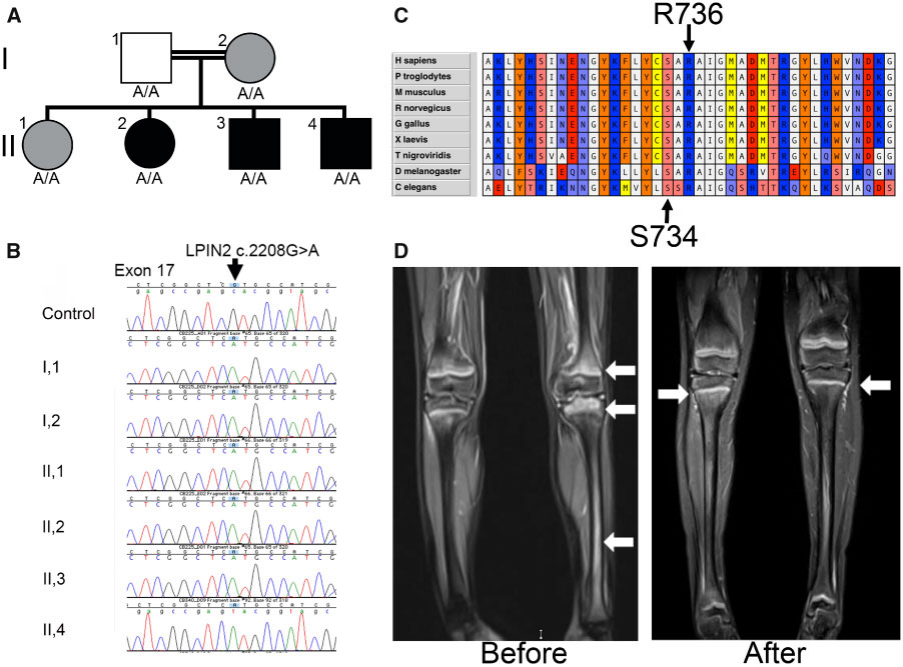
Family affected by Majeed syndrome (**A)** Pedigree, the genotype of *LPIN2* c.2208 is shown under each individual. Black symbols indicate severely affected individuals, grey symbols represent mildly affected individuals and the white symbol indicates the father, who manifested no clinical symptoms. (**B**) Chromatograms from a control sample (top) and family members as indicated (left), all showing homozygosity for the c.2208 G > A missense change in exon 17 of *LPIN2*. (**C)** Alignment of lipin-2 amino acid sequence from a variety of species showing the R736 residue altered in the family reported here is conserved to the nematode *C. elegans*, indicating ∼600 million years of evolutionary conservation. This region of the protein is highly conserved, and residue S734 is also indicated for comparison as this is an established cause of Majeed syndrome. (**D)** MRI images showing inflammation in the affected limb and knee joint of the patient before (arrows show active osteitis) and after (arrows show resolving residual osteitis) treatment with anakinra.

We describe a consanguineous Pakistani family where the index child presented in infancy with a conglomeration of features indicative of possible Majeed syndrome: failure to thrive, recurrent fevers, irritability, limb pains, osteitis on whole body MRI, severe microcytic anaemia (CDA on bone marrow examination) and high inflammatory markers (CRP 110 mg/L, ESR 90 mm/h). An extensive immunology/infectious diseases workup was inconclusive or normal. The proband’s DNA was screened using the Oxford Red Cell Panel [5], which includes ∼50 genes implicated in inherited anaemias, including *LPIN2*, which revealed a homozygous G > A transition in *LPIN2* (c.G2207A) leading to replacement of a highly conserved arginine at position 736 with histidine (p.R736H) (Fig. 1B). Further questioning revealed the mother and elder sister had both suffered milder forms of anaemia with limb pains requiring only NSAIDs. The proband’s two brothers, mother and father were found to be homozygous for this variant (Fig. 1C). R736H is predicted to be pathogenic by Polyphen2 (with a probability of 1) and is present in only 6 out of 245 402 alleles in the Gnomad database (http://gnomad.broadinstitute.org/). Arginine 736 lies in a highly conserved region of lipin-2 and is two amino acids away from the previously reported pathogenic S734L change (Fig. 1C), and both missense changes affect buried residues, likely affecting stability of this region.

Initial management for mild intermittent osteitis involved NSAIDs and infrequent oral corticosteroids to which there was partial transient response. At the age of 6, due to ongoing symptomatic osteitis (Fig. 1D), a therapeutic trial of bisphosphonates (3 monthly intravenous pamidronate) as for sporadic CNO was initiated. Transient improvement in bone pain (partial improvement of bone lesions on MRI scan) was observed with a slight improvement in inflammatory markers.

Due to ongoing nocturnal bone pain, new lesions on MRI with synovitis adjacent to bone lesions, intermittent high inflammatory markers (ESR highest 190, CRP highest 134, Hb lowest 69) and poor growth (0.4th centile for weight and height), IL-1 blockade with daily subcutaneous anakinra (1 mg/kg) was commenced. This resulted in resolution of bone pain, significant improvement of bone lesions on MRI scan, normalization of inflammatory markers (ESR 2, CRP 0.2, Hb 107), improved appetite with weight gain, improved sleep and school attendance, and significant improvement in CHAQ and pain visual analog score scores, and negated the need for regular NSAIDs.

Since then, the proband’s two younger brothers have developed severe bone pains (with confirmed osteitis by MRI), moderately high inflammatory markers and chronic microcytic anaemia, responding favourably to anakinra, clinically behaving like the proband rather than the mother or elder sister. The father has been asymptomatic apart from non-specific knee pains. We characterized the *LPIN2* locus in this family and found it to be identical over the extent of the gene, suggesting the loci are identical by descent and the phenotypic variability is unlikely to originate from segregation of an alternative *LPIN2* allele.

There is no clear genotype/phenotype correlation between the severity of the genetic change and degree of anaemia, and identical mutations can lead to widely different phenotypes [1, 2, 6], suggesting the influence of modifying factors. Clues to this may be obtained by comparing the transcriptional response in cells from differentially affected patients.

The protein encoded by *LPIN2*, lipin-2, is a magnesium-dependent phosphotidate phosphatase (peroxidase–antiperoxidase) catalysing the conversion of phosphatidic acid to diacylglycerol, a key step in lipid metabolism. The previously reported S734L variant affects only the peroxidase–antiperoxidase activity of lipin-2 [7] and the close proximity of the R736H mutation reported here means it may have a similar effect. Given the involvement of IL-1 in Majeed syndrome, it strongly suggests impaired lipid metabolism increases IL-1 production. Mechanistically, impaired lipin-2 peroxidase–antiperoxidase function could increase inflammasome activity and therefore increase IL-1 production through a failure to preserve the proper lipid environment. Such an environment is required by the purinergic receptor P2X_7_R to maintain cellular potassium levels that thereby prevent inflammasome assembly [8]. Further work is required to explain the link with microcytic anaemia.

In conclusion we report a novel *LPIN2* mutation causative of Majeed syndrome with widely variable expressivity and penetrance in one consanguineous family and no evidence of skin involvement. Although there was partial response to bisphosphonates in our patients, we propose use of IL-1 blockade as evidenced by the sustained improvement with anakinra. IL-1 blockade whether by anakinra or canakinumab in Majeed syndrome is supported by recent evidence of the role of lipin-2 in the inflammasome and highlights the key role of IL-1 signalling in Majeed syndrome. Finally, due to the variable penetrance and expressivity, we propose that *LPIN2* be included individually in gene panels for both microcytic anaemia and bone autoinflammatory disease where genetic cause is indicated by a young age of onset, evidence of high levels of systemic inflammation, additional features such as fevers, skin lesions consanguinity or a positive family history consistent with CRMO or a lack of response to therapy with bisphosphonates.

*Funding:* This work was supported by a Medical Research Council grant (number MC_uu_12009) and by the National Institute for Health Research (NIHR) Oxford Biomedical Research Centre (BRC). The views expressed are those of the author(s) and not necessarily those of the NHS, the NIHR or the Department of Health.

*Disclosure statement:* The authors have declared no conflicts of interest.
